# Supplementary material for: DMPC Phospholipid Bilayer as a Potential Interface for Human Cystatin C Oligomerization: Analysis of Protein-Liposome Interactions Using NMR Spectroscopy
Source: Membranes (Basel). 2020 Dec 24;11(1):13. doi: 10.3390/membranes11010013 (PMC7824490; doi:10.3390/membranes11010013)
Supplement: Supplementary file 1 [file membranes-11-00013-s001.pdf]

Supplementary Materials: DMPC Phospholipid Bilayer as a Potential Interface for Human Cystatin C Oligomerization: Analysis of Protein-Liposome Interactions Using NMR Spectroscopy

Przemyslaw Jurczak [1], Kosma Szutkowski [2], Slawomir Lach [1], Stefan Jurga [2], Paulina Czaplewska [3], Aneta Szymanska [1] and Igor Zhukov [4,*]

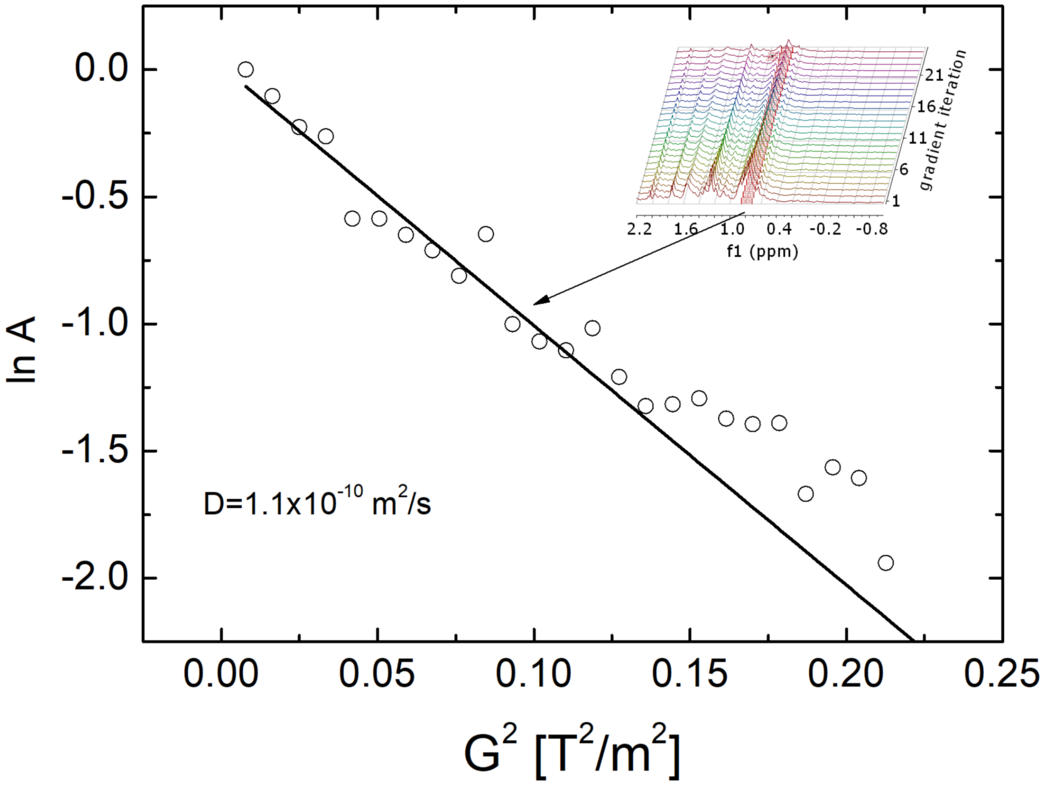

Figure S1. Analysis of the translational diffusion data acquired on 18.8 T at 298 K with PGSE-NMR technique. The faster diffusion process, observed on the graph, appear due to the presence of some amount of unfolded protein.
